# The 90th anniversary of *Angiostrongylus cantonensis*: from local discovery to global endemic

**DOI:** 10.1186/s40249-025-01384-8

**Published:** 2025-11-12

**Authors:** Shan Lv, Xiao-Nong Zhou

**Affiliations:** 1 National Institute of Parasitic Diseases at Chinese Center for Disease Control and Prevention (Chinese Center for Tropical Diseases Research); Key Laboratory on Parasite and Vector Biology, National Health Commission; WHO Collaborating Centre for Tropical Diseases; National Center for International Research on Tropical Diseases, Ministry of Science and Technology, Shanghai, People’s Republic of China; 2 School of Global Health, Chinese Center for Tropical Diseases Research, Shanghai Jiao Tong University School of Medicine, Shanghai, People’s Republic of China; 3 Hainan Center for Tropical Diseases Research (Hainan Sub-Center of Chinese Center for Tropical Diseases Research), Haikou, People’s Republic of China

Ninety years ago, in the bustling port city of Guangzhou (Canton), a nematode entered the annals of science, its discovery was recorded in a local journal in 1933 [1], and then Professor H.T. Chen named and described the nematode as *Pulmonema cantonensis* in a French journal in 1935 [2]. Today, *Angiostrongylus cantonensis*, the rat lungworm, stands as a global health challenge. Eosinophilic meningitis due to *A. cantonensis* infection, also known as angiostrongyliasis, is a paradigm of emerging infectious diseases in the world. The disease is a stark reminder of the intricate, often perilous, connections between human, animal, and ecosystem health. The journey of *A. cantonensis* from a localized rodent parasite to a worldwide cause of eosinophilic meningitis is a compelling narrative of scientific discovery, epidemiological expansion, and the urgent need for a unified, transdisciplinary response. The 90th anniversary of its discovery provides a pivotal moment to reflect on its past, critically assess its present impact, and chart a course for the future through the lens of the One Health approach.

This editorial will trace the historical trajectory of *A. cantonensis*, from its initial identification by Professor H.T. Chen to its current status as an endemic pathogen across tropical and subtropical regions. We will explore the key factors driving its global spread, including globalization, climate change, and evolving human behaviors. Drawing extensively on the Chinese experience as a case study, we will illustrate the dynamic nature of this emerging zoonosis and the evolution of national response strategies. Our discussion will focus on the indispensable role of the One Health paradigm, not merely as a conceptual framework but as a practical, actionable strategy for surveillance, prevention, and control. Finally, we will outline critical research priorities that must be addressed to mitigate the burden of angiostrongyliasis and prevent it from becoming a significant pandemic threat. The story of the rat lungworm is more than a parasitological curiosity; it is a powerful allegory for our interconnected world and a call to action for global health solidarity.

## Historical discovery and early research: a Cantonese origin story

The scientific saga of *A. cantonensis* begins with the meticulous work of Professor H.T. Chen, a pioneering Chinese parasitologist whose academic pedigree was as impressive as his scientific acumen. Having earned his M.Sc. from the University of Minnesota and a Ph.D. from Harvard University in the late 1920s, Chen returned to China with a wealth of knowledge and a keen eye for discovery. In 1933, he published his initial findings on a novel nematode infecting rats in a local English-language journal, though at that time, he refrained from formally naming the organism within the field of zoology [1]. This early observation was the first spark in what would become a 90-year-long investigation.

The formal nomenclatural act came two years later in 1935. In a publication in the French journal titled *Annales de Parasitologie Humaine et Comparée*, Chen meticulously described and named the parasite as *Pulmonema cantonensis*, honoring its geographical origin in Canton (now Guangzhou)[2]. His detailed morphological description laid the foundation for all subsequent taxonomic work. Two years later, a Japanese scientist reported a new nematode named as *Hemastrongylus ratti* [3]. Now it is known as the synonym of *A. cantonensis*. For years following its discovery, *A. cantonensis* was considered a parasite of veterinary interest, primarily a curiosity affecting the pulmonary arteries of its definitive rodent hosts, particularly rats of the genus *Rattus*.

The mid-twentieth century witnessed a gradual shift in this perception. The true zoonotic potential of the rat lungworm was horrifically unveiled in 1944 in Taiwan Province of China. Dr. Nomura and Dr. Lin reported a case of eosinophilic meningitis in a 15-year-old boy, marking the first documented human infection [4]. This landmark case report opened a new chapter in the parasite’s history, transforming it from an obscure animal pathogen into a direct threat to human health. The 1950s and 1960s saw a flurry of research activity, primarily driven by researchers in Hawaii of USA, Australia and Southeast Asia, who began to piece together the parasite’s complex and fascinating life cycle [5, 6].

Humans are accidental, dead-end hosts in this cycle of transmission. Humans become infected by consuming raw or undercooked infected mollusks or paratenic hosts, or by consuming contaminated products (e.g., lettuce) that contain small snails or slugs [7]. Once ingested, the L3 larvae follow a similar path to that in rats, migrating to the central nervous system. However, in humans, the larvae are unable to complete their development. They die in the brain and spinal cord, triggering a severe inflammatory reaction, called eosinophilic meningitis or meningoencephalitis, that is the hallmark of neuroangiostrongyliasis. This understanding of the life cycle and pathogenesis was crucial for developing diagnostic and prevention strategies. The discovery of related species, such as *A. mackerrasae* in Australia and *A. malaysiensis* in Malaysia, further complicated the epidemiological picture and highlighted the diversity within the *Angiostrongylus* genus [8, 9].

## A global journey: epidemiological expansion and endemicity

From its presumed origin in Southern China, *A. cantonensis* has embarked on a remarkable journey of global expansion, transforming from a regionally endemic parasite into a pathogen of worldwide concern. By the 1970s, its endemicity was firmly established across Southeast Asia and the Pacific Islands, including Thailand, Vietnam, the Philippines, Indonesia, Papua New Guinea, and Hawaii of USA [6]. These regions, with their warm, humid climates and culinary traditions involving raw mollusks, provided the perfect ecological and cultural niche for the parasite to thrive.

**Fig. 1:**
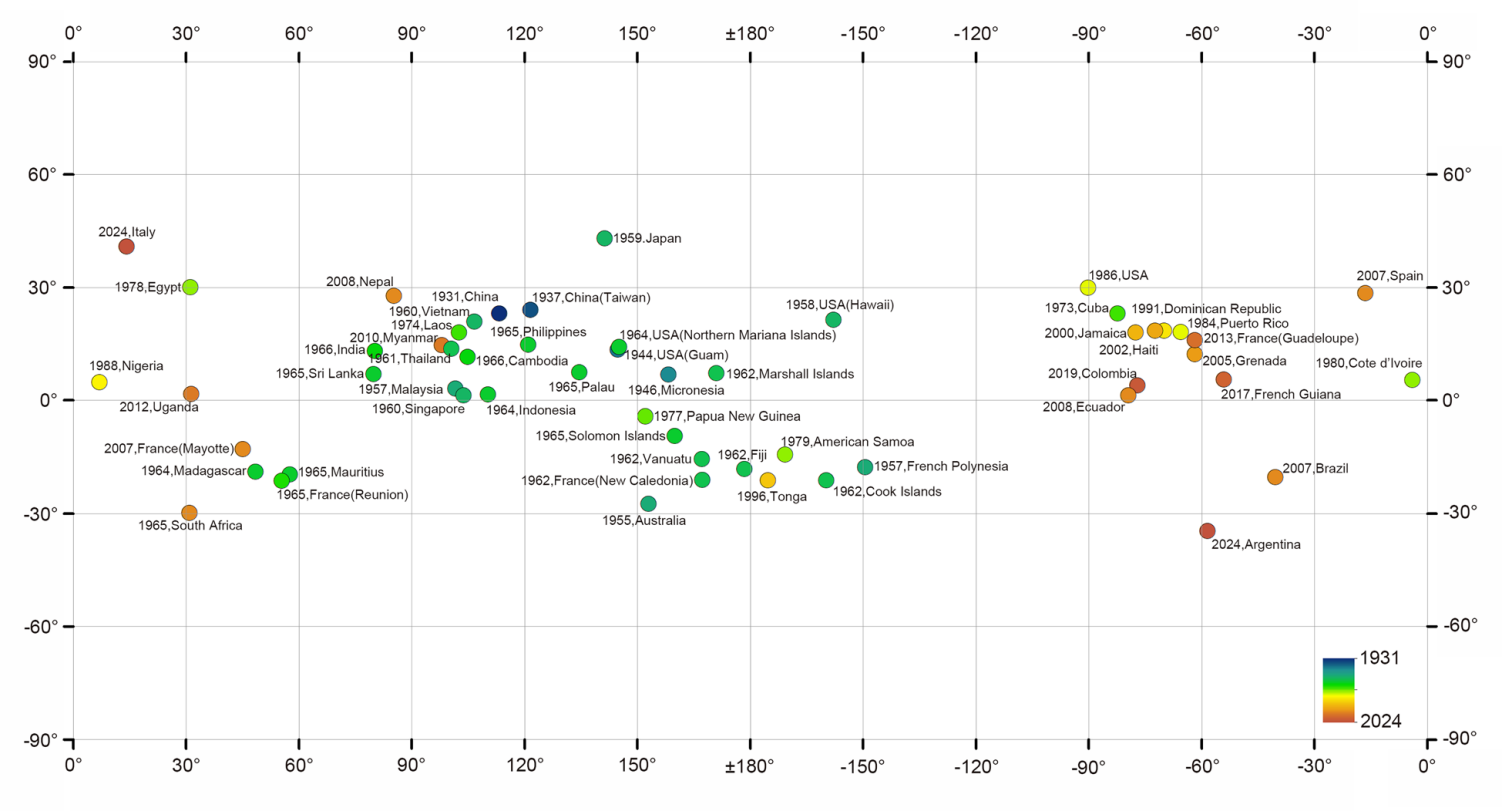
The range expansion of *Angiostrongylus cantonesnsis*. The important places with name and the year for the first presence of the rat lungworm are denoted by color, classified by the discovery year

The late 20th and early twenty-first centuries have witnessed an alarming expansion of its geographic range (Fig. 1). The parasite was reported in Africa (e.g., Egypt and Nigeria), the Americas (e.g., Southern USA, Brazil, and the Caribbean countries), and even in Europe (e.g., Spain and Italy). This expansion is not random but driven by a confluence of powerful anthropogenic and environmental factors, such as climate changes.

(I) Globalization and trade: The international movement of goods is a primary vector for the parasite’s spread. The invasive giant African land snail (*Achatina fulica*) and various species of *Pomacea* spp. (apple snails), both highly efficient intermediate hosts, have been transported globally through the pet trade, as a food source, and accidentally via cargo shipments. These snails, once established in new environments, can readily acquire the parasite from local rat populations, creating new transmission cycles. The outbreak of angiostrongyliasis in Beijing in 2006, for instance, was definitively linked to *Pomacea* spp. snails imported from southern China[10].
(II) Climate change: Rising global temperatures and altered precipitation patterns are expanding the suitable habitats for both the definitive rodent hosts and the intermediate mollusk hosts. Regions previously too cold for the establishment of parasite’s life cycle are becoming increasingly permissive. Modeling studies suggest that climate change will continue to facilitate its spread into higher latitudes and altitudes, potentially exposing millions of new people to the risk of infection[11].
(III) Human behavior and cultural practices: The consumption of raw or undercooked intermediate or paratenic hosts remains the most significant risk factor for human infection. Dishes like raw snail salad, prawns, or frogs, and even the accidental ingestion of small slugs on unwashed vegetables, are common transmission routes. The growing popularity of “exotic” cuisines and ecotourism may inadvertently expose naive populations to the parasite.
(IV) Urbanization and synanthropic rodents: The proliferation of urban environments has led to a boom in populations of synanthropic rodents, e.g., rats that live in close association with humans. The rats act as a persistent reservoir for the parasite, ensuring its survival and transmission even in densely populated cities.

With the development of molecular phylogenetics, more research have provided compelling evidence for this expansion. Studies, such as the one by Tian et al. [12], reveal a clear pattern that higher genetic diversity is observed in Southeast Asia, the presumed ancestral range, while newly colonized regions like the Americas and Pacific islands exhibit significantly lower diversity. This genetic bottleneck strongly supports the “Asian origin hypothesis” and suggests multiple, separate introduction events. For example, the establishment of the parasite in the Pacific region is thought to have occurred during World War II through troop movements, while its introduction to the Americas likely occurred via separate trade routes [12]. The genetic structure also shows distinct differences between eastern and western populations, and the related species *A. malaysiensis* has been found to expand beyond its traditional home range, further complicating the global epidemiological landscape.

## The Chinese experience: a case study in an emerging zoonosis

China’s history with *A. cantonensis* serves as a powerful microcosm of the global challenge posed by this emerging zoonosis. The country’s experience, marked by initial cases, devastating outbreaks, and the development of a robust national response, offers invaluable lessons for other nations facing similar threats.

The first confirmed human case of angiostrongyliasis in Chinese mainland was documented in 1984 [13]. A 13-year-old boy from Xuwen, Guangdong Province, presented with symptoms of meningitis. A critical epidemiological link was established: the boy frequently helped his family feed ducks by crushing *Achatina fulica* snails. Examination of his cerebrospinal fluid (CSF) revealed the presence of *A. cantonensis* larvae, confirming the diagnosis. Fortunately, the boy made a full recovery. This case was a watershed moment, demonstrating that the parasite was not just a theoretical risk but an active public health issue in China.

The first fatal case, reported in 1995 from Guangzhou, underscored the potential severity of the disease. An 11-month-old infant from a poor residential area died from the infection. Post-mortem examination revealed hundreds of adult worms in her pulmonary arteries, and infected *A. fulica* snails were found in the vicinity of her home[14]. This tragic case highlighted the vulnerability of children and the role of poor sanitation and environmental contamination in the disease transmission.

The first major outbreak occurred in 1997 in Wenzhou, Zhejiang Province, a region north of the traditional endemic zones in southern China. A total of 182 clients at a single restaurant were at risk. Of the 105 individuals who consumed a raw snail dish, 47 were diagnosed with angiostrongyliasis [15]. Crucially, none of the 77 clients who ate only cooked snails developed symptoms, providing strong evidence for the transmission route. A clear dose–response relationship was observed: the incidence was 86.2% among those who ate more than four pieces of snail, compared to 28.9% among those who ate less. Investigation confirmed the presence of the parasite in both the restaurant’s *Pomacea* spp. snails and local rats [16]. This outbreak was a wake-up call, demonstrating the potential for large-scale foodborne transmission and the parasite’s ability to spread to new regions within the country.

The largest outbreak to date in China occurred in Beijing, the capital, in 2006. Between June and August, 160 cases were registered, with 100 requiring hospitalizations. This outbreak was particularly significant because Beijing is not a traditional endemic area due to lack of *Pomacea* spp. snail habitats. Nearly all hospitalized patients confirmed consuming snail dishes at two branches of the same restaurant. Again, the investigation traced the source to infected *Pomacea* spp. snails imported from southern China [17]. This event, occurring in the capital city of the country, triggered a massive public health response and became the catalyst for a comprehensive national program to combat angiostrongyliasis, one of the food-borne infectious diseases.

The launch of the national program in 2006 marked a turning point, with the following key initiatives:

- *First national survey (2006–2007):* A systematic investigation on the distribution of *A. cantonensis* and its rodent and mollusk hosts across China [10].
- *Standardized diagnosis (2010):* The Ministry of Health released official diagnostic criteria for angiostrongyliasis, ensuring uniformity in case detection and reporting.
- *Routine surveillance (2009–present):* A national surveillance and reporting system was established to monitor human cases and snails in markets, in some areas, environmental contamination.
- *Capacity building:* Ongoing efforts to train healthcare professionals in diagnosis and management and to strengthen laboratory and research capabilities related to control and prevention of angiostrongyliasis.

## One Health: a unifying approach for a complex problem

The story of *A. cantonensis* indicates why the “One Health” approach is not just beneficial but essential. The parasite’s life cycle inherently links the health of humans, animals (both domestic and wild), and the shared environment. Its emergence and spread are driven by human activities that disrupt ecological balances. Therefore, any attempt to control it must be equally integrated and involve cross-sectoral cooperation.

As defined by the One Health high-level expert panel (OHHLEP), supported by the Quadripartite organizations including Food and Agriculture Organization of the United Nations (FAO), World Health Organization (WHO), United Nations Environment Programme (UNEP), World Organisation for Animal Health (WOAH), One Health is an integrated, unifying approach that aims to sustainably balance and optimize the health of people, animals and ecosystems [18]. This definition moves beyond collaboration to true integration, recognizing that the health of each domain is inextricably linked to the others.

The application of One Health approach to the control of *A. cantonensis* transmission involves several key pillars:

1. *Integrated surveillance-response systems:* The core of a One Health strategy is the breaking down of silos between human health, animal health, and environmental sectors. For *A. cantonensis*, this means creating a system where data on human meningitis, veterinary neuroangiostrongyliasis (a significant issue in dogs, horses, and wildlife), and environmental monitoring of mollusk and rat populations are collected, shared, and analyzed in real-time. This integrated system allows us to detect the threats at the animal-environment interface before they spill over to humans, enabling a rapid, coordinated response.
2. *Multi-sectoral cooperation:* Effective implementation requires governance structures that facilitate collaboration between sectors of health, agriculture, and environment. The International Health Regulations (IHR) for human health and the Performance of Veterinary Services (PVS) standards for animal health provide frameworks that can be harmonized under a One Health governance (OHG) umbrella. The Quadripartite’s One Health Joint Plan of Action (OH JPA) (2022–2026) operationalizes this, with thematic groups focused on implementation, surveillance, and understanding the drivers of spillover [19].
3. *Intra-disciplinary research:* One Health demands research that transcends traditional disciplinary boundaries. This includes ecologists studying mollusk population dynamics, climatologists modeling the impact of climate change on parasite distribution, social scientists understanding the cultural drivers of raw food consumption, and economists evaluating the cost–benefit of interventions. The life cycle of *A. cantonensis* is a perfect illustration of the human-animal-ecosystem interface, where interventions can be targeted at multiple points: rodent control, mollusk management, food safety regulations, and public health education.
4. *Environmental integration: *A truly effective One Health approach must embed environmental considerations at its core. This means moving beyond a focus on just human and animal health to actively manage the ecosystems in which they exist. For *A. cantonensis*, this involves strategies like precision intervention in hotspots, as demonstrated in Hainan, Shanghai, and Yunnan, China, which combine mollusk control, rodent eradication, and environmental monitoring. It also means recognizing the role of biodiversity loss in increasing the risk of zoonotic spillover, a concept increasingly central to the One Health approach.

The value proposition of One Health is clear. The World Bank estimated net annual benefits between USD 4 billion and USD 35 billion from its implementation, driven by reduced pandemic risk, improved public health, and more efficient health systems [20]. For a disease like angiostrongyliasis, which sits at the confluence of trade, environment, food, and health, One Health is not just the best approach but is the only viable path forward.

## Research priorities for the next decade: charting the future course

As we commemorate 90 years since the discovery of *A. cantonensis*, we should look forward with a clear and ambitious research agenda. While significant progress has been made, critical knowledge gaps remain. Addressing these will be essential for achieving sustainable control and preventing the continued global spread of this insidious parasite. We propose key priorities to guide the scientific community over the next decade.

1. *Comprehensive geophylogenetic and population genomic studies:* While the Asian origin of *A. cantonensis* is well-supported, the fine-scale details of its global dispersal remain incomplete. We need systematic, large-scale nuclear and mitochondrial genomic studies focusing on understudied but potentially critical regions like the Philippines, Indonesia, Myanmar, and India. Such research can help resolve questions about the number and timing of introduction events to the Americas, Africa and Europe. This requires building local sequencing capacity and fostering international sample-sharing networks.
2. *Global vulnerability and risk mapping:* The parasite’s range is expanding, but we lack a comprehensive, dynamic global map of risk. We need to develop and refine predictive models that integrate multiple data layers: including climate projections (temperature, rainfall), land use change, global shipping and trade routes, species distribution models for key hosts (e.g., rats, *Achatina* spp., *Pomacea* spp.), and human socioeconomic factors. This would allow public health officials to identify emerging hotspots, target surveillance efforts proactively, and inform quarantine and biosecurity policies.
3. *Establishment of an integrated system on health, veterinary and wildlife surveillance:* The impact of *A. cantonensis* on animal health is a significant, yet often overlooked, aspect of its epidemiology. Neuroangiostrongyliasis is a serious and often fatal disease in companion animals (especially dogs), livestock, and wildlife in endemic areas, like Australia and the USA [21, 22]. Establishing robust surveillance systems for animal cases would serve a dual purpose. First, it would address the direct animal health and welfare concerns. Second, and critically from a One Health perspective, it would act as an early warning system for human risk [23, 24]. An increase in canine cases in a specific area, for example, could signal heightened environmental contamination and predict a subsequent rise in human cases. This requires integrating veterinary diagnostic labs into national surveillance networks and promoting awareness among veterinarians and public health professionals.
4. *Enhanced diagnostic accuracy and species identification:* The *Angiostrongylus* genus contains several morphologically similar species. Misidentification in previous studies and case reports is a real possibility, which can confound our understanding of species-specific distributions, host ranges, and pathogenicity. We strongly recommend that DNA sequencing become a standard component of species identification, alongside traditional morphology, for both larvae recovered from patients and adult worms from hosts. This will ensure the accuracy of epidemiological data and improve our understanding of the relative public health importance of different *Angiostrongylus* species.

## Conclusion: a call for united action

The 90-year journey of *A. cantonensis* from a single local discovery to a global public health concern is a powerful narrative of our modern world. It is a story of scientific curiosity, of a parasite’s remarkable adaptability, and of the profound consequences of our interconnectedness. The rat lungworm is more than just a pathogen; it is a sentinel, signaling the health of our ecosystems and the vulnerabilities in our global systems.

Today we stand at a crossroad. We either persist with a fragmented response that lets *A. cantonensis* continue its silent spread, or seize this moment to unite for control and prevent *A. cantonensis* infections. We call upon researchers, clinicians, veterinarians, ecologists, policymakers, and community leaders to forge a new, collaborative path. By working together — united as a storm, driven by a norm, backed by One Health — we can not only mitigate the burden of angiostrongyliasis but also build a more resilient and healthier world for all.
